# Virulence gene landscapes of *Salmonella* in Eastern and Southern Africa

**DOI:** 10.3389/fmicb.2025.1631550

**Published:** 2025-07-11

**Authors:** Mohamed-Yousif Ibrahim Mohamed, Ihab Habib

**Affiliations:** ^1^Department of Veterinary Medicine, College of Agriculture and Veterinary Medicine, United Arab Emirates University, Al Ain, United Arab Emirates; ^2^ASPIRE Research Institute for Food Security in the Drylands (ARIFSID), United Arab Emirates University, Al Ain, United Arab Emirates

**Keywords:** *Salmonella*, virulence genes, food chain, foodborne infection, East and Southern Africa

## Abstract

Salmonellosis is one of the main foodborne diseases in Eastern and Southern Africa, however its different forms are not fully understood. Based on studies conducted over 20 years, the review discusses how *invA*, the *spv* operon, the *cdtB*-*pltAB* typhoid toxin cassette, the adhesion factor *bapA*, and loci related to stress responses (*pagC, mgtB*) affect pathogenic strains isolated from livestock, wildlife, produce, and humans from various countries. Findings reveal pronounced ecological and geographic variation, S. Typhimurium and S. Enteritidis in Ethiopia's dairy chain and Tanzanian backyard poultry carry *spv* at rates exceeding 80%, while whole-genome studies from South Africa document the continent's most extensive accessory-gene repertoires and identify fully virulent strains in reptiles and market vegetables. Human outbreaks mirror this diversity, Nairobi pediatric isolates harbor universal *hilA*/*sopB* and *Stn*; Ugandan epidemics rely on chromosomal factors despite minimal *spvB*; Rwandan Moero serovars uniquely possess the cytolethal-distending-toxin cassette. Altogether, the data suggests a significant need for syncing genomic disease surveillance with the One-Health approach, this will allow for early detection of hybrid and migrating bacteria, shielding children, serious disease sufferers, and those serving the food sector against more spread of dangerous pathogens.

## 1 Introduction

Health authorities worldwide continue to consider *Salmonella* as a major public health threat that causes 93.8 million foodborne infections with around 150,000 deaths annually each year. Such infections stand as major contributors to global expenses from foodborne diseases (EFSA., 2019; ECDC EFSA., 2022). Foodborne diseases persist considerably across Africa because of weak food safety structures that face regulatory limitations and outdated facilities. Grasping the factors that cause *Salmonella* throughout the food supply chain enables the creation of successful prevention measures (WHO, 2015). *Salmonella* contamination throughout Africa creates substantial healthcare risks for the population which result in recorded outbreaks that lead to hospital admissions and death alongside economic expenses (Ibrahim et al., 2018; Elafify et al., 2022). The lack of available resources and inadequate law enforcement continues to make complete control measures difficult which demonstrating the importance of establishing joint regional monitoring and support operations (Habib and Mohamed, 2022; Teklemariam et al., 2023).

*Salmonella enterica* is the main pathogenic member of the *Enterobacteriaceae* family and is one of the global leaders in causing bacterial gastroenteritis. Salmonellosis remains a serious public health concern across different European nations (Al-Gallas et al., 2022). The cellular invasion and survival of *Salmonella* bacteria inside macrophages represent an essential pathogenic process since it allows the bacteria to escape host immunity while remaining inside the host (Pinedo et al., 2022). The intracellular lifestyle of these bacteria creates diagnostic and therapeutic challenges because traditional treatment methods often fail to eliminate bacteria from intracellular reservoirs. Targeted intervention strategies aimed at intracellular survival mechanisms are essential to achieve effective outcomes for clinical capabilities and public health management of salmonellosis.

Bacterial virulence-related genes present in *Salmonella* efficiently initiate and accelerate the development of foodborne illnesses. The genetic elements give the pathogen the ability to adhere to host cells while breach tissues while escaping immune detection leading to increased infection potential (Vilela et al., 2020). The investigation of these genes represents a necessary step for the development of specific interventions to control *Salmonella* outbreaks and their prevention efforts. The need to grasp the molecular sequences that regulate bacterial aggression together with environmental stress paths is highlighted by such investigative work (Mohamed et al., 2022).

Experimental animal studies confirm *Salmonella* strains with specific virulence genes responsible for cellular attachment including intra-cellular survival present more hazardous infections, resulting in higher mortality than virulence-deficient strains (Khalefa et al., 2021; Hull et al., 2022; Mohamed et al., 2024). *Salmonella* Pathogenicity Islands (SPIs) genomic clusters increase experimental infection risks and lead to severe disease manifestations (Kombade and Kaur, 2021). Scientific studies of clinical microbial isolates have proven the existence of correlations between particular virulence marker occurrences and human cases of salmonellosis severity (Wang et al., 2020; Borah et al., 2022). The occurrence of the *stn* gene which produces enterotoxin is associated with higher hospitalization rates and more severe clinical expressions in patients (Nikiema et al., 2021).

Advances in whole-genome sequencing (WGS), a method that determines the complete DNA sequence of an organism's genome at a single time, have facilitated detailed analysis of the genetic features that underpin *Salmonella* virulence. By examining the complete genomes of various isolates, scientists have been able to map the distribution and frequency of virulence genes across different strains, shedding light on their role in disease severity and immune evasion (Mohamed et al., 2025). The genomic findings validate previous research demonstrating that particular genetic patterns relate to infections that spread deeply into the body and resist treatment (Nikiema et al., 2021). Additionally, functional genomics approaches including gene knockouts and expression analysis in both *in vitro* and *ex vivo* models have proven instrumental in clarifying the specific contributions of individual virulence genes to pathogenesis. These experimental frameworks not only validate the importance of these genes in promoting *Salmonella* infection but also help assess their role in determining clinical outcomes (Lozano-Villegas et al., 2023).

Some genetic virulence components missing in *Salmonella* isolates from food sources diminish their ability to produce clinical salmonellosis (Wang et al., 2020). The virulence genes produce proteins that help bacteria establish residence and invade host cells. These microbe strains become less pathogenic because of their absence or reduced expression levels of essential virulence genes (Vilela et al., 2020). Ingestion of bacteria with lower virulence often results in mild or no apparent symptoms in human bodies. Organisms that lack necessary virulence factors demonstrate a reduced ability to spread in human digestive tracts which decreases the chance of infections after contact with contaminated material (Wang et al., 2020). The health risk potential of foodborne *Salmonella* strains originates from the virulence genes that they contain or lack. Detecting genetic markers serves vital functions for both danger evaluation in public health and intervention development for foodborne infection control (Habib et al., 2023b; Oueslati et al., 2023).

The pathogenic capabilities of *Salmonella* species result from virulence genes that exist as *Salmonella* Pathogenicity Islands (SPIs) throughout the bacterial chromosome (Dougnon et al., 2017). The five classified SPIs provide essential knowledge to scientists and SPI-1 along with SPI-2 stand out because they encode the Type III secretion systems (T3SSs) (Cerny and Holden, 2019; Lerminiaux et al., 2020). The interaction between SPI-1 and SPI-2 demonstrates different functions because SPI-1 enables cell invasion and triggers inflammation whereas SPI-2 drives phagocytic cell survival across the body (Wemyss and Pearson, 2019). The genetic construct *invA* within SPI-1 exists across all *Salmonella* strains because it serves as the critical factor for host cell penetration. The *spiC* gene encoded by SPI-2 produces essential secretion system components required for virulence while operating independently from flagellar structures (Hasan, 2021; Wang et al., 2021). Both SPI-3 and SPI-4 exist throughout all *Salmonella* lineages however, the patterns of occurrence for SPI-4 and SPI-5 remain uncertain (Wang et al., 2020). SPI-4 contributes to early interactions with intestinal epithelial cells and supports long-term colonization, including the *orfL* gene linked to survival within macrophages (Albanwawy and Abdul-Lateef, 2021). SPI-5 is involved in multiple stages of the infection process, with *pipD* playing a notable role (Wang et al., 2020). Additionally, *Salmonella* harbors extra-chromosomal virulence determinants such as the *Salmonella* virulence plasmid (*spvRABCD*), which enhances systemic dissemination and enables replication at extraintestinal sites (Dougnon et al., 2017; Hsu et al., 2019). Polyamines, which are present in elevated concentrations in various fermented, aged, and plant-derived foods, serve critical functions in cellular homeostasis and microbial viability. In the context of foodborne pathogens such as *Salmonella*, elevated dietary polyamine levels may enhance bacterial resilience within the gastrointestinal tract, potentially contributing to heightened virulence and persistence during infection. This association suggests that polyamines may play a significant role in modulating pathogen-host interactions. Therefore, elucidating the link between polyamine concentrations in food and microbial pathogenicity is essential for informing targeted strategies aimed at mitigating foodborne illnesses and safeguarding public health (Mohamed et al., 2019a; Krysenko and Wohlleben, 2022).

## 2 Overview of virulence determinants in Eastern and Southern Africa

### 2.1 Materials and methods

This study utilized a narrative review approach to synthesize findings related to *Salmonella* virulence genes in East and Southern Africa. A comprehensive literature search was conducted using electronic databases such as PubMed (https://pubmed.ncbi.nlm.nih.gov/) and Google Scholar (https://scholar.google.com/) to identify relevant studies published over the last two decades (Paré et al., 2015). Only peer-reviewed materials maintained scientific rigor for the review process so non-peer-reviewed pieces such as opinion writing letters to the editor or anecdotal documentation were excluded. The search was refined using specific keywords per country “*Salmonella* virulence genes Ethiopia.” Studies were included if they reported on the virulence gene profiles of *Salmonella* isolates from food, environmental, or human sources within African countries. Articles that lacked relevant data or failed to meet inclusion criteria were excluded from the review (Paré et al., 2015).

### 2.2 Virulence factors in Eastern and Southern Africa

In Eastern and Southern Africa, salmonellosis causes real concerns for public health, especially when hot weather blackouts, and shortages of water, favor bacterial persistence and foodborne transmission (Mohamed et al., 2019b; Mohamed, 2024). Because they come into contact with animals, raw meat, and dirty food, veterinarians, livestock and poultry farmers, employees at slaughterhouses, traders of live chickens, market butchers, and house-food handlers are more likely to contract diseases (Mohamed and Habib, 2023). These observations set the stage for understanding how various virulence genes in Salmonella contribute to its transmission and impact in this region. Even though the threat is real, there are very few country-specific studies on *Salmonella* infections in humans in lands as far apart as Burundi and South Africa over the last 20 years, so research is needed for each region to guide prevention steps.

Building on this context, Table 1 compiles the virulence factors found in *Salmonella enterica* taken from foods, livestock, wildlife, and humans in Eastern and Southern Africa. The *invA* invasion gene, found only in those bacteria that can enter cells, was found in 80–100% of the strains in Ethiopia, Kenya, Tanzania, Botswana, and South Africa (Munuo et al., 2022; Beyene et al., 2024; Bywater et al., 2024; Webale, 2024). In addition, the *spv* operon located on plasmids (*spvABCRD*) supports the bacteria's systemic movement. Over 83% of the S. Typhimurium and S. Enteritidis were studied in Ethiopian dairy (Beyene et al., 2024) and over 81% of the S. Enteritidis from Tanzanian backyard poultry had the gene, but the same was negative for other serovars like S. Ball or S. Blockley (Rukambile et al., 2021). The *spv* was found in 14% of animals but 62% of human clinical samples in routine conditions and remained at 100% during the outbreak.

**Table 1:** Prevalence of virulence genes in *Salmonella* serotypes isolated from food and human sources in East and Southern Africa.

**Regional distribution**	**Source of samples (food or human)**	**Temporal trends**	***Salmonella* serotypes (total number)**	Virulence genes (%)^*^	**Methods for detecting virulence genes**	**References**
**East Africa**						
**Ethiopia**						
Northwest part of Ethiopia	Sources from dairy supply chain and associated regions	June 2022 to August 2023	Uganda (*n* = 11)	*invA* (100)*, spvC* (0)	PCR technique	Beyene et al., 2024
			*S. enterica* subsp. *Diarizonae* (*n* = 7)	*invA* (100)*, spvC* (0)		
			Typhimurium (*n* = 6)	*invA* (100)*, spvC* (83.3)		
			Bredeney (*n* = 2)	*invA* (100)*, spvC* (0)		
			Enteritidis (*n* = 1)	*invA* (100)*, spvC* (100)		
			Urbana (*n* = 1)	*invA* (100)*, spvC* (0)		
Southern Ethiopia	Raw milk samples		*Salmonella* isolates (*n =* 40)	*invA* (80)	PCR technique	Gebeyehu et al., 2022
**Kenya**						
Nairobi city	Diarrheic children under 5 years	2024	*Salmonella* isolates (*n =* 9)	*invA* (100)*, hila* (100)*, sopB* (100)*, Stn* (100)	PCR technique	Webale, 2024
Rural Western Kenya	Clinical *Salmonella* enterica	February 2004 to June 2005	*Salmonella typhi* isolates (*n =* 3)	*invA* (0), *spvA* (33.3), *spvB* (33.3), *spvC* (33.3), *spvD* (33.3), *spvR* (33.3)	PCR technique	Onyango et al., 2010
**Rwanda**						
Northern Province of Rwanda	Animal	2019	Typhimurium (*n* = 1)	*spvA* (100)*, spvB* (100)*, spvC* (100)*, spvD* (100)*, spvR* (100)	Whole-genome sequencing (WGS)	Byukusenge, 2019
	Humans		Moero (*n =* 3)	*cdtB* (100)*, pltA* (100)*, pltB* (100)		
**Tanzania**						
Morogoro, Tanzania	Chicken	October 2019 and May 2021	*Salmonella* isolates (*n =* 11)	*invA* (100)*, iroB* (100)	PCR technique	Munuo et al., 2022
Rural Central Tanzania	Chicken	2021	Typhimurium (*n =* 1)	*spvB* (100)*, spvC* (100)*, spvR* (100)	Whole-genome sequencing (WGS)	Rukambile et al., 2021
			Enteritidis/Gallinurum (*n =* 1)	*spvB* (100)*, spvC* (100)*, spvR* (0)		
			Ball (*n =* 4)	*spvB* (0)*, spvC* (0)*, spvR* (0)		
			Haardt/Blockley (*n =* 1)	*spvB* (0)*, spvC* (0)*, spvR* (0)		
			Braenderup (*n =* 1)	*spvB* (0)*, spvC* (0)*, spvR* (0)		
Kibong'oto Infectious Diseases Hospital	Clinical *Salmonella* enterica	June 2019	Typhimurium (*n =* 8)	*invA* (50)*, spvC* (37.5)	PCR technique	Mkangara et al., 2020
**Uganda**						
Mulago National Referral Hospital, Tororo Hospital, and Kasese District	Clinical *Salmonella* enterica	Between 2007 and 2009	*Salmonella* isolates (*n =* 25)	*spvB* (80)*, spiA* (88)*, pagC* (92)*, msgA* (92)*, sipB* (84)*, spaN* (92)	PCR technique	Kagirita et al., 2017
	Human epidemic		*Salmonella* isolates (*n =* 23)	*spvB* (4.3)*, spiA* (82.6)*, pagC* (82.6)*, msgA* (95.6)*, sipB* (87)*, spaN* (95.6)		
	Cattle		*Salmonella* isolates (*n =* 7)	*spvB* (14.3)*, spiA* (85.7)*, pagC* (85.7)*, msgA* (85.7)*, sipB* (85.7)*, spaN* (85.7)		
	Pigs		*Salmonella* isolates (*n =* 2)	*spvB* (0)*, spiA* (50)*, pagC* (50)*, msgA* (100)*, sipB* (100)*, spaN* (100)		
	Poultry		*Salmonella* isolates (*n =* 12)	*spvB* (0)*, spiA* (66.7)*, pagC* (66.7)*, msgA* (75)*, sipB* (83.3)*, spaN* (83.3)		
**Southern Africa**						
**Botswana**						
Northern Botswana in Chobe District	Vegetables obtained from retail markets	2022	*Salmonella* isolates (*n =* 7)	*invA* (100)	PCR technique	Bywater et al., 2024
**Malawi**						
Blantyre, Malawi	Queen Elizabeth Hospital	2024	Typhimurium (*n =* 1)	*spvA* (0)*, spvB* (0)*, spvC* (0)*, spvD* (100)*, macB* (100)	Whole-genome sequencing (WGS)	Kumwenda et al., 2024
**South Africa**						
Mahikeng city of North West Province, South Africa	Healthy broiler chickens from chicken abattoirs	2024	Typhimurium and S. Enteritidis (*n =* 22)	*hilA* (100)*, ssrB* (100)*, pagC* (100), 1)*, bapA* (36.4)*, sopB* (31.8)*, marT* (22.7)*, vexA* (18.2)*, nlpI* (18.2)*, oafA* (13.6)*, cdtB* (27.3)*, spvB* (18.2)*, pagN* (0)	PCR technique	Ramatla et al., 2024
Gauteng Province, South Africa	Chickens sold at the informal chicken market	2023	*Salmonella* isolates (*n =* 157)	*invA* (100)*, spiC* (91.7)*, shdA* (87.8)*, mgtB* (83.4)*, sopE* (77.7) *pefC* (0.6)*, sefC* (2.5)	PCR technique	Mokgophi et al., 2024
Limpopo Province, South Africa	Wild Reptiles	2023	*Salmonella* isolates (*n =* 30)	*pagN* (100)*, hilA* (96.7)*, ssrB* (96.7)*, prgH* (86.7)*, marT* (86.7)	PCR technique	Mlangeni et al., 2024
Mafikeng, South Africa	Poultry farms	2019	*Salmonella* isolates (*n =* 46)	*invA* (100)*, spy* (39)*, hilA* (9)*, misL* (30)*, sdfI* (13)*, orfL* (11)*, spiC* (9)	PCR technique	Ramatla et al., 2020
South Coast in South Africa	Animals	2018	*Salmonella* isolates (*n =* 106)	*invA* (100)*, iroB* (30.2)*, pipD* (62.3)*, spiC* (18.9)*, int1* (34.9)	PCR technique	Mthembu et al., 2019
Eastern Cape, South Africa	Patients with diarrhea, Nelson Mandela Academic Hospital Complex (NAMHC)	2011	*Salmonella* isolates (*n =* 119)	*invA* (88.2)*, fliC* (12.6)	PCR technique	Bisi-Johnson et al., 2011
**Zimbabwe**						
Zimbabwe	Human	2014	*Salmonella* isolates (*n =* 13)	*Spv* (61.5)	PCR technique	Farai, 2014
	Animal		*Salmonella* isolates (*n =* 36)	*Spv* (13.9)		
Selected locations of Zimbabwe	Human (outbreak)	2012	*Salmonella* isolates (*n =* 8)	*Spv* (100)	PCR technique	Nhidza et al., 2012
	Animals		*Salmonella* isolates (*n =* 32)	*Spv* (37.5)		

^*^The percentage (%) of Salmonella serotypes is calculated from the positive samples (isolated target bacteria).

While *invA* and *spv* are central to virulence, regional genome sequencing efforts have uncovered broader profiles. Whole-genome studies from South Africa have discovered the greatest number of additional genes in the region (Mlangeni et al., 2024; Ramatla et al., 2024). Many industrial broilers contained the adhesion gene *bapA*, enterotoxin *sopB*, typhoid-toxin cassette genes *cdtB*-*pltAB*, gut stress-resistance genes *pagC*, and stress-response gene *mgtB*, whereas backyard flocks had these features at lesser and more variable levels (Ramatla et al., 2020; Mlangeni et al., 2024). Researchers found that Limpopo reptiles carried a lot of the type-III secretion regulator *prgH* (Mlangeni et al., 2024), while Botswana's market vegetables hosted only virulent lead-containing *invA*, both supporting the significance of including fresh products in One-Health tracking (Bywater et al., 2024).

Studies have found that *Salmonella* strains causing cases or outbreaks in humans share many genes that allow them to attack, spread within the body, and release toxic compounds. The gene core invasion *invA* was found in all of Nairobi's pediatric diarrhea isolates by Webale, (2024) and in South Africa 88% by Bisi-Johnson et al. (2011), however a decade earlier in rural Kenya, it was unexpectedly absent from S. Typhi (Onyango et al., 2010). *Salmonella* regulator *hilA* and effector *sopB* were also detected in 100% of bacteria in Webale's Kenyan cohort, hinting at strong SPI-1 invasion of current non-typhoidal strains (Webale, 2024). Plasmid-supported *spv* genes that promote intracellular growth and infection in the blood were rare or not detected in Nairobi, but were prevalent in Zimbabwe (Nhidza et al., 2012; Farai, 2014), some regions of western Kenya (Onyango et al., 2010), and hospital samples from Tanzania (Rukambile et al., 2021). In line with this, epidemic strains in Uganda lack the *spvB*, indicating that they survive through a combination of chromosomal genetic factors (over 80% for all of them) (Kagirita et al., 2017).

Further, a similar variation was found in the *ctdt* for the cytolethal distending toxin, which appeared exclusively in Rwanda's Moero (Byukusenge, 2019), while *Stn* was found in every Nairobi sample (Webale, 2024). Lastly, the flagellar gene *fliC* was present in only 13% of South African samples (Bisi-Johnson et al., 2011), which agrees with the expectation that bacteria living in the airways can avoid immune responses by losing their flagella. These findings reinforce the notion that the distribution and function of virulence genes vary significantly across the region and must be interpreted within local ecological and host contexts. Hence, the combination of these trends reveals that virulence is affected by the host population, and local conditions, and monitoring of the *Salmonella* genome is important to predict changes to plasmid-mediated virulence and to guide local control efforts.

Surveillance, however, remains fragmented and heavily reliant on single-gene PCR panels that may overlook emerging hybrid pathotypes or misclassify partial plasmid variants (e.g., *spvD*-only isolates from Malawi) (Kumwenda et al., 2024). It is important to use WGS routinely since it can provide in-depth information on virulence, track how plasmids spread, and detect any serovars linked to typhoid toxins in non-typhoid bacteria (Mohamed et al., 2024). A unified WGS strategy would also resolve concerns about exchanging regional products, including South African eggs in Uganda's markets and Tanzanian beef entering Malawi, direct intervention programs for poultry in Malawi, improvements to salad greens cold storage in Botswana, and supervising plasmids for both Malawi and Zimbabwe (Habib et al., 2023a).

Overall, it is clear that *invA* functions as a unique identifier for African *Salmonella*. Still, the presence of plasmids and pathogenicity islands heavily affects the sickness profile and is linked to the host, habitat, and how much is produced in African countries. As seen with Campylobacter in the Gulf region, young children, people whose immune systems do not work properly, and groups of workers are especially at risk from *Salmonella*. This highlights the need for integrated, cross-sectoral surveillance that links human, animal, food, and environmental data using advanced genomic tools. Integrating surveillance for humans, animals, food, and environments with the help of WGS and well-equipped and skilled laboratories is required to handle the growing issues related to invasive and foodborne salmonellosis in Eastern and Southern Africa.

## 3 Conclusions

This review noted that *Salmonella enterica*'s infection mechanisms in Eastern and Southern Africa are driven primarily by the common *invA* gene, as well as by different secondary factors such as the plasmid-borne *spv* operon, typhoid toxin genes, adhesion proteins such as *bapA*, and stress-related loci such as *pagC* and *mgtB*, among others. High carriage rates of *spv* in Ethiopia's dairy chain, Tanzanian backyard poultry, and Zimbabwean outbreak strains underscore its pivotal role in systemic disease. Whole-genome data from South Africa reveal even broader repertoires that vary with production intensity and ecological niche. Fresh-produced isolates in Botswana and reptile reservoirs in Limpopo further illustrate how fully virulent strains move beyond traditional livestock pathways. Nonetheless, surveillance is not complete since using single-gene PCR panels fails to detect hybrids and certain plasmid fragments. This finding suggests these isolates have other, yet unidentified, ways to cause disease. By applying these results, researchers should regularly keep watch over the genetic makeup, monitor the sharing of plasmid resistance, and prepare different strategies to address it. For this reason, knowledge of the source is vital for planning steps like improving the cold chain and reptile management.

## References

[B1] Albanwawy J. N. A. Abdul-Lateef L. A. (2021). Molecular Detection of Some of the Salmonella typhi Virulence Genes Isolated in the Province of Babylon/Iraq. Available online at: http://annalsofrscb.ro (Accessed March 10, 2025).

[B2] Al-Gallas N. Belghouthi K. Barratt N. A. Ghedira K. Hotzel H. Tomaso H.. (2022). Identification and characterization of multidrug-resistant ESBL-producing *Salmonella enterica* serovars Kentucky and Typhimurium isolated in Tunisia CTX-M-61/TEM-34, a novel cefotaxime-hydrolysing β-lactamase of *Salmonella*. J. Appl. Microbiol. 132, 279–289. 10.1111/jam.15211 34252258

[B3] Beyene A. M. Alemie Y. Gizachew M. Yousef A. E. Dessalegn B. Bitew A. B.. (2024). Serovars, virulence factors, and antimicrobial resistance profile of non-typhoidal *Salmonella* in the human-dairy interface in Northwest Ethiopia: a one health approach. PLoS Negl. Trop. Dis. 18:e0012646. 10.1371/journal.pntd.0012646 39565761 PMC11578527

[B4] Bisi-Johnson M. A. Obi C. L. Vasaikar S. D. Baba K. A. Hattori T. (2011). Molecular basis of virulence in clinical isolates of *Escherichia coli* and *Salmonella* species from a tertiary hospital in the Eastern Cape, South Africa. Gut Pathog. 3:9. 10.1186/1757-4749-3-9 21663681 PMC3125331

[B5] Borah P. Dutta R. Das L. Hazarika G. Choudhury M. Deka N. K.. (2022). Prevalence, antimicrobial resistance and virulence genes of *Salmonella* serovars isolated from humans and animals. Vet. Res. Commun. 46, 799–810. 10.1007/s11259-022-09900-z 35167002

[B6] Byukusenge M. (2019). Descriptive Epidemiology and Comparative Genomic Analysis of Nontyphoidal Salmonella serotypes From Rwanda. New York, NY: The Pennsylvania State University.

[B7] Bywater A. Dintwe G. Alexander K. A. Ponder M. A. (2024). Characterization of diarrheagenic *Escherichia coli* and *Salmonella enterica* from produce in the Chobe district of Botswana. J. Food Prot. 87:100351. 10.1016/j.jfp.2024.100351 39187132

[B8] Cerny O. Holden D. W. (2019). *Salmonella* SPI-2 type III secretion system-dependent inhibition of antigen presentation and T cell function. Immunol. Lett. 215, 35–39. 10.1016/j.imlet.2019.01.006 30771380

[B9] Dougnon T. V. Legba B. Deguenon E. Hounmanou G. Agbankpe J. Amadou A.. (2017). Pathogenicity, epidemiology and virulence factors of *Salmonella* species: a review. Not. Sci. Biol. 9, 460–466. 10.15835/nsb9410125

[B10] ECDC and EFSA. (2022). The European Union summary report on antimicrobial resistance in zoonotic and indicator bacteria from humans, animals and food in 2019–2020. EFSA J. 20:e07209. 10.2903/j.efsa.2022.7209 35382452 PMC8961508

[B11] EFSA. (2019). The European Union One Health 2018 Zoonoses report. EFSA J. 17:e05926. 10.2903/j.efsa.2019.5926 32626211 PMC7055727

[B12] Elafify M. Darwish W. S. El-Toukhy M. Badawy B. M. Mohamed R. E. Shata R. R. (2022). Prevalence of multidrug resistant *Salmonella* spp. in dairy products with the evaluation of the inhibitory effects of ascorbic acid, pomegranate peel extract, and D-tryptophan against *Salmonella* growth in cheese. Int. J. Food Microbiol. 364:109534. 10.1016/j.ijfoodmicro.2022.109534 35033976

[B13] Farai N. A. (2014). Molecular Typing of Salmonella Serotypes Isolated From Wildlife, Domesticated Animal Species and Humans in Zimbabwe. Harare: University of Zimbabwe.

[B14] Gebeyehu A. Taye M. Abebe R. (2022). Isolation, molecular detection and antimicrobial susceptibility profile of *Salmonella* from raw cow milk collected from dairy farms and households in southern Ethiopia. BMC Microbiol. 22:84. 10.1186/s12866-022-02504-2 35361106 PMC8969351

[B15] Habib I. Elbediwi M. Mohteshamuddin K. Mohamed M.-Y. I. Lakshmi G. B. Abdalla A.. (2023a). Genomic profiling of extended-spectrum β-lactamase-producing *Escherichia coli* from pets in the United Arab Emirates: unveiling colistin resistance mediated by mcr-1.1 and its probable transmission from chicken meat – a One Health perspective. J. Infect. Public Health 16, 163–171. 10.1016/j.jiph.2023.10.034 37957104

[B16] Habib I. Mohamed M. Y. I. (2022). “Foodborne infections in the Middle East,” in Food Safety in the Middle East (Elsevier), 71–107. 10.1016/B978-0-12-822417-5.00005-2

[B17] Habib I. Mohteshamuddin K. Mohamed M.-Y. I. Lakshmi G. B. Abdalla A. Bakhit Ali Alkaabi A. (2023b). Domestic pets in the United Arab Emirates as reservoirs for antibiotic-resistant bacteria: a comprehensive analysis of extended-spectrum beta-lactamase producing *Escherichia coli* prevalence and risk factors. Animals 13:1587. 10.3390/ani13101587 37238016 PMC10215154

[B18] Hasan T. O. (2021). Genomic Diversity Analysis of Salmonella spp. From Broiler and Layer Flocks and their Feed and Water in Karbala, Iraq. Baghdad: University of Baghdad.

[B19] Hsu C.-H. Li C. Hoffmann M. McDermott P. Abbott J. Ayers S.. (2019). Comparative genomic analysis of virulence, antimicrobial resistance, and plasmid profiles of *Salmonella* Dublin isolated from sick cattle, retail beef, and humans in the United States. Microb. Drug Resist. 25, 1238–1249. 10.1089/mdr.2019.0045 31149890 PMC11555760

[B20] Hull D. M. Erin H. Lyndy H. Siddhartha T. (2022). Multidrug resistance and virulence genes carried by mobile genomic elements in *Salmonella enterica* isolated from live food animals, processed, and retail meat in North Carolina, 2018–2019. Int. J. Food Microbiol. 2:109821. 10.1016/j.ijfoodmicro.2022.109821 35816956

[B21] Ibrahim M. J. Abdul-Aziz S. Bitrus A. A. Mohammed D. G. Abu J. Bejo S. K.. (2018). Occurrence of multidrug resistant (MDR) *Campylobacter* species isolated from retail chicken meats in Selangor, Malaysia and their associated risk factors. Malays. J. Microbiol. 14, 261–272. 10.21161/mjm.107717

[B22] Kagirita A. A. Baguma A. Owalla T. J. Bazira J. Majalija S. (2017). Molecular characterization of *Salmonella* from human and animal origins in Uganda. Int. J. Bacteriol. 2017, 1–9. 10.1155/2017/4604789 28634597 PMC5467339

[B23] Khalefa H. S. Ahmed Z. S. Abdel-Kader F. Ismail E. M. Elshafiee E. A. (2021). Sequencing and phylogenetic analysis of the stn gene of *Salmonella* species isolated from different environmental sources at Lake Qarun protectorate: the role of migratory birds and public health importance. Vet. World 14, 2764–2772. 10.14202/vetworld.2021.2764-2772 34903938 PMC8654765

[B24] Kombade S. Kaur N. (2021). “Pathogenicity island in *Salmonella*,” in Salmonella spp. - A Global Challenge (London: IntechOpen). 10.5772/intechopen.96443

[B25] Krysenko S. Wohlleben W. (2022). Polyamine and ethanolamine metabolism in bacteria as an important component of nitrogen assimilation for survival and pathogenicity. Med. Sci. 10:40. 10.3390/medsci10030040 35997332 PMC9397018

[B26] Kumwenda B. Canals R. Predeus A. V. Zhu X. Kröger C. Pulford C.. (2024). *Salmonella enterica* serovar Typhimurium ST313 sublineage 2.2 has emerged in Malawi with a characteristic gene expression signature and a fitness advantage. Microlife 5:uqae005. 10.1093/femsml/uqae005 38623411 PMC11018118

[B27] Lerminiaux N. A. MacKenzie K. D. Cameron A. D. S. (2020). *Salmonella* pathogenicity island 1 (SPI-1): the evolution and stabilization of a core genomic type three secretion system. Microorganisms 8:576. 10.3390/microorganisms8040576 32316180 PMC7232297

[B28] Lozano-Villegas K. J. Herrera-Sánchez M. P. Beltrán-Martínez M. A. Cárdenas-Moscoso S. Rondón-Barragán I. S. (2023). Molecular detection of virulence factors in *Salmonella* serovars isolated from poultry and human samples. Vet. Med. Int. 2023, 1–9. 10.1155/2023/1875253 36910894 PMC9998162

[B29] Mkangara M. Mbega E. R. Chacha M. (2020). Molecular identification of *Salmonella* Typhimurium from village chickens based on invA and spvC genes. Vet. World 13, 764–767. 10.14202/vetworld.2020.764-767 32546923 PMC7245706

[B30] Mlangeni L. N. Ramatla T. Lekota K. E. Price C. Thekisoe O. Weldon C. (2024). Occurrence, antimicrobial resistance, and virulence profiles of *Salmonella* serovars isolated from wild reptiles in South Africa. Int. J. Microbiol. 2024:5213895. 10.1155/2024/5213895 38222969 PMC10787053

[B31] Mohamed M.-Y. I. (2024). Campylobacteriosis in North Africa. AIMS Agric. Food 9, 801–821. 10.3934/agrfood.2024043

[B32] Mohamed M.-Y. I. Abdul-Aziz S. Jalila A. Khairani-Bejo S. Puan C. L. Bitrus A. A.. (2019a). Occurrence of antibiotic resistant *Campylobacter* in wild birds and poultry. Malays. J. Microbiol. 15, 143–151. 10.21161/mjm.180096

[B33] Mohamed M.-Y. I. Abu J. Abdul-Aziz S. Zakaria Z. Rashid A. Awad E. A. (2019b). Occurrence of antibiotic resistant *C. jejuni* and *E. coli* in wild birds, chickens, environment and humans from Orang Asli Villages in Sungai Siput, Perak, Malaysia. Am. J. Anim. Vet. Sci. 14, 158–169. 10.3844/ajavsp.2019.158.169

[B34] Mohamed M.-Y. I. Abu J. Aziz S. A. Zakaria Z. Khan A. R. Habib I. (2022). Occurrence of antibiotic resistant *C. jejuni* and *E. coli* in wild birds, chickens, humans, and the environment in Malay villages, Kedah, Malaysia. Vet. Med. 67, 298–308. 10.17221/102/2021-VETMED 39100641 PMC11296224

[B35] Mohamed M.-Y. I. Habib I. (2023). Pathogenic *E. coli* in the food chain across the Arab countries: a descriptive review. Foods 12:3726. 10.3390/foods12203726 37893619 PMC10606471

[B36] Mohamed M.-Y. I. Habib I. Khalifa H. O. (2024). *Salmonella* in the food chain within the Gulf Cooperation Council countries. AIMS Microbiol. 10, 468–488. 10.3934/microbiol.2024023 39219759 PMC11362266

[B37] Mohamed M.-Y. I. Khalifa H. O. Habib I. (2025). Food pathways of *Salmonella* and its ability to cause gastroenteritis in North Africa. Foods 14:253. 10.3390/foods14020253 39856919 PMC11765101

[B38] Mokgophi T. M. Gcebe N. Fasina F. Adesiyun A. A. (2024). Molecular characterization of virulence and resistance genes in *Salmonella* strains isolated from chickens sold at the informal chicken market in Gauteng Province, South Africa. J. Food Saf. 44:e13110. 10.1111/jfs.13110

[B39] Mthembu T. P. Zishiri O. T. El Zowalaty M. E. (2019). Detection and molecular identification of *Salmonella* virulence genes in livestock production systems in South Africa. Pathogens 8:124. 10.3390/pathogens8030124 31405078 PMC6789496

[B40] Munuo L. A. Katakweba A. A. Lyimo B. M. Muhairwa A. P. (2022). Prevalence, characterization and antimicrobial resistance profiles of *Salmonella* isolates from healthy broiler and free-range chickens in Morogoro, Tanzania. Tanzan. J. Health Res. 23, 1–14. 10.4314/thrb.v23i1.6

[B41] Nhidza A. F. Saidi B. Makaya P. V. Kanyoka P. Sithole-Niang I. Savadye D. (2012). Distribution of SPV genes, plasmid profiles and pulsotypes of *Salmonella enteritidis* isolates of animal and human origins in selected locations of Zimbabwe. J. Clin. Med. Res. 4, 63–74. 10.5897/JCMR11.02838147025

[B42] Nikiema M. E. M. Kakou-ngazoa S. Ky/Ba A. Sylla A. Bako E. Addablah A. Y. A.. (2021). Characterization of virulence factors of *Salmonella* isolated from human stools and street food in urban areas of Burkina Faso. BMC Microbiol. 21:338. 10.1186/s12866-021-02398-6 34895140 PMC8665542

[B43] Onyango D. M. Kakai R. Nyandago W. E. Ghebremedhin B. Konig W. Kong B. (2010). Integron-plasmid mediated antibiotic resistance and virulence factors in clinical *Salmonella enterica* serovars in rural Western Kenya. Afr. J. Pharm. Pharmacol. 4, 490–497.

[B44] Oueslati W. Ridha Rjeibi M. Benyedem H. Jebali M. Souissi F. Selmi R.. (2023). Serotype occurrence, virulence profiles, antimicrobial resistance and molecular characterization of *Salmonella* isolated from hospitalized patients with gastroenteritis in great Tunisia between 2010 and 2020. Antibiotics 12:526. 10.3390/antibiotics12030526 36978394 PMC10044041

[B45] Paré G. Trudel M. C. Jaana M. Kitsiou S. (2015). Synthesizing information systems knowledge: a typology of literature reviews. Inform. Manag. 52, 183–199. 10.1016/j.im.2014.08.008

[B46] Pinedo C. L. Mughini-Gras L. Franz E. Hald T. Pires S. M. (2022). Sources and trends of human salmonellosis in Europe, 2015–2019: an analysis of outbreak data. Int. J. Food Microbiol. 379:109850. 10.1016/j.ijfoodmicro.2022.109850 35961158

[B47] Ramatla T. Khasapane N. G. Mlangeni L. N. Mokgokong P. Ramaili T. Ndou R.. (2024). Detection of *Salmonella* pathogenicity islands and antimicrobial-resistant genes in *Salmonella enterica* serovars enteritidis and Typhimurium isolated from broiler chickens. Antibiotics 13:458. 10.3390/antibiotics13050458 38786186 PMC11117945

[B48] Ramatla T. A. Mphuthi N. Ramaili T. Taioe M. O. Thekisoe O. M. M. Syakalima M. (2020). Molecular detection of virulence genes in *Salmonella* spp. isolated from chicken faeces in Mafikeng, South Africa. J. S. Afr. Vet. Assoc. 91, 1–7. 10.4102/jsava.v91i0.1994 32787420 PMC7433231

[B49] Rukambile E. Sintchenko V. Muscatello G. Wang Q. Kiiru J. Maulaga W.. (2021). *Campylobacter* and *Salmonella* in scavenging indigenous chickens in rural Central Tanzania: prevalence, antimicrobial resistance, and genomic features. Microbiol. Res. 12, 440–454. 10.3390/microbiolres12020030

[B50] Teklemariam A. D. Al-Hindi R. R. Albiheyri R. S. Alharbi M. G. Alghamdi M. A. Filimban A. A. R.. (2023). Human salmonellosis: a continuous global threat in the farm-to-fork food safety continuum. Foods 12:1756. 10.3390/foods12091756 37174295 PMC10178548

[B51] Vilela F. P. dos Prazeres Rodrigues D. Costa R. G. Casas M. R. T. Falcão J. P. Campioni F. (2020). High similarity and high frequency of virulence genes among *Salmonella* Dublin strains isolated over a 33-year period in Brazil. Braz. J. Microbiol. 51, 497–509. 10.1007/s42770-019-00156-5 31701384 PMC7203401

[B52] Wang M. Qazi I. H. Wang L. Zhou G. Han H. (2020). *Salmonella* virulence and immune escape. Microorganisms 8:407. 10.3390/microorganisms8030407 32183199 PMC7143636

[B53] Wang Y. Liu G. Zhang J. Gu D. Hu M. Zhang Y.. (2021). WbaP is required for swarm motility and intramacrophage multiplication of *Salmonella* Enteritidis *spiC* mutant by glucose use ability. Microbiol. Res. 245:126686. 10.1016/j.micres.2020.126686 33429286

[B54] Webale M. K. (2024). Genotyping virulence and resistance profiles in *Salmonella* isolated from diarrheic children in Nairobi city, Kenya. Gastroenterol. Hepatol. Bed Bench 17, 430–437. 10.22037/ghfbb.v17i4.3026 40406434 PMC12094503

[B55] Wemyss M. A. Pearson J. S. (2019). Host cell death responses to non-typhoidal *Salmonella* infection. Front. Immunol. 10:1758. 10.3389/fimmu.2019.01758 31402916 PMC6676415

[B56] WHO. (2015). WHO Estimates of the Global Burden of Foodborne Diseases: Foodborne Disease Bur- den Epidemiology Reference Group 2007–2015. Geneva, Switzerland: World Health Organization.

